# Identification of *Theileria* spp. in sheep and goats from Jeddah, Saudi Arabia, using molecular techniques

**DOI:** 10.7717/peerj.12596

**Published:** 2021-12-10

**Authors:** Dina M. Metwally, Reem Alajmi, Muslimah N. Alsulami, Isra M. Al-Turaiki, Rewaida Abdel-Gaber, Afrah F. Alkhuriji, Haleema H. Albohiri, Khalil Mohamed, Hanadi B. Baghdadi, Manal F. El-Khadragy, Guillermo T. Isaias, Saeed El-Ashram

**Affiliations:** 1 Department of Parasitology, Faculty of Veterinary Medicine, Zagazig University, Zagazig, Egypt; 2 Department of Zoology, Faculty of Science, King Saud University, Riyadh, Saudi Arabia; 3 Department of Biology, College of Science, University of Jeddah, Jeddah, Saudi Arabia; 4 Department of Information Technology, College of Computer and Information Sciences, King Saud University, Riyadh, Saudi Arabia; 5 Zoology Department, Faculty of Science, Cairo University, Cairo, Egypt; 6 Epidemioligy Department, Faculty of Public Health and Health Informatics, Umm Al-Qura University, Mecca, Saudi Arabia; 7 Biology Department, College of Science, Imam Abdulrahman Bin Faisal University, Dammam, Saudi Arabia; 8 Basic and Applied Scientific Research Center, Imam Abdulrahman Bin Faisal University, Dammam City, Saudi Arabia; 9 Department of Biology, Faculty of Science, Princess Nourah Bint Abdulrahman University, Riyadh, Saudi Arabia; 10 Department of Zoology and Entomology, Faculty of Science, University of Helwan, Cairo, Egypt; 11 Department of Poultry Science, University of Arkansas, Fayetteville, AR, USA; 12 Faculty of Science, Kafrelsheikh University, Kafr el-Sheikh, Egypt; 13 College of Life Science and Engineering, Foshan University, Foshan, Guangdong Province, China

**Keywords:** *Theileria* spp., Polymerase chain reaction (PCR), *18S rRNA*

## Abstract

**Background:**

Thileriosis is a tick -born disease caused by hemoprotozoan parasites which has global veterinary and economic implications.

**Methods:**

Blood samples were collected from 216 sheep and 83 goats from Jeddah, Saudi Arabia, were analyzed to determine whether the animals were infected with *Theileria* spp. parasites. The parasites were detected using a polymerase chain reaction (PCR) targeting the gene of *18S rRNA* followed by sequencing.

**Results:**

According to obtained findings, *Theileria* spp. were detected in sheep (57.8%, 48/83) and goats (51.9%, 112/216). Phylogenetic analysis to sequence data showed that *T. ovis* identified in this study were found to be closely connected to an isolate from Turkey, with 84.4–99.8% pairwise identity and 52.35–99.79% coverage.

## Introduction

In developing countries, sheep and goats are important livestock species. However, successful livestock production is hampered by a variety of factors, such as mismanagement, infestation by ectoparasites, contagious infectious diseases, and nutritional deficiencies (Tomley & Shirley, 2009). Tick-borne diseases are a major cause of inefficiency (Alanazi et al., 2019). Ticks are regarded as key transmitting agents in tropical and subtropical regions for ruminant piroplasmosis (Brites-Neto, Duarte & Martins, 2015). This hemoprotozoan infection is caused by the genera *Theileria* and *Babesia* (Mehlhorn & Schein, 1985) and has been reported in studies of small ruminants in Saudi Arabia (Al-Khalifa et al., 2009; El-Azazy, El-Metenawy & Wassef, 2001; Ghandour, Tahir & Shalaby, 1989; Hussein et al., 1991; Mostafa & Saad, 2014).

Theileriosis in small ruminants is caused by protozoa from the genus *Theileria* transmitted by ixodid ticks of the genera *Rhipicephalus*, *Hyalomma*, *Amblyomma,* and *Haemaphysalis* (Mamatha et al., 2017). In 1914, ovine theileriosis was first reported in Egypt in Sudanese sheep (Littlewood, 1957). Similar diseases were later reported in various regions of China (Guo et al., 2002), Northern Spain (Nagore et al., 2004), Iran (Razmi et al., 2006), Pakistan (Durrani et al., 2011), Turkey (Altay, Dumanli & Aktas, 2012), and Ethiopia (Gebrekidan et al., 2014). The clinical signs of theileriosis include anemia, icterus, enlargement of superficial lymph nodes, and hemoglobinuria, which ultimately result in death and loss of production (Hassan, Raoofi & Lotfollahzadah, 2015).

There are currently six *Theileria* spp. associated with ovine theileriosis; *Theileria lestoquardi*, *T. luwenshuni,* and *T. uilenbergi* are highly pathogenic (Schnittger et al., 2000), while *T. separata*, *T. ovis,* and *T. recondite* are less pathogenic and cause subclinical diseases in sheep and goats (Ahmed, 2006). Malignant theileriosis in cattle and or sheep has been recorded in Turkey (Sayin et al., 1997), Iran (Hashemi-Fesharki, 1997), Iraq (Latif, Hewa & Bakir, 1977), Saudi Arabia (El-Metenawy, 1999; Hussein et al., 1991), and Oman (Tageldin et al., 2005).

Acute-phase theileriosis is traditionally identified using Giemsa-stained thin blood smears and clinical signs (Zakkyeh et al., 2012). However, in some cases, recovered animals sustain subclinical infections that are not microscopically visible (Rahmani-Varmale, Tavassoli & Esmaeilnejad, 2019). Molecular approaches are highly sensitive and specific, enabling the genotypic identification of many hemoparasites (Mamatha et al., 2017; Metwally et al., 2021). Polymerase chain reaction (PCR) gives us short-term perspective for piroplasm detection (Kubelová, Mazancová & Široký, 2012). The genetic markers of mitochondrial cytochrome c oxidase I (*COX I*), *16S rRNA* (Zahler et al., 1997), and *18S rRNA* (Ranjbar-Bahadori et al., 2012) were used for identification and classification of Theileria and Babesia parasites.

Few molecular studies were available in Saudi Arabia about tick-borne pathogens (Alanazi et al., 2018a; Alanazi et al., 2018b; Alanazi et al., 2020; Alanazi et al., 2021a; Alanazi et al., 2021b; Zakham et al., 2021). Therefore, the present study used the genetic marker of *18S rRNA* to identify and classify piroplasmosis in sheep and goat blood samples.

## Materials and Methods

### Ethical statement

This study was approved by the Deanship of Postgraduate Studies, University of Jeddah, Saudi Arabia (UJ-212430001).

### Study area

**Figure 1:**
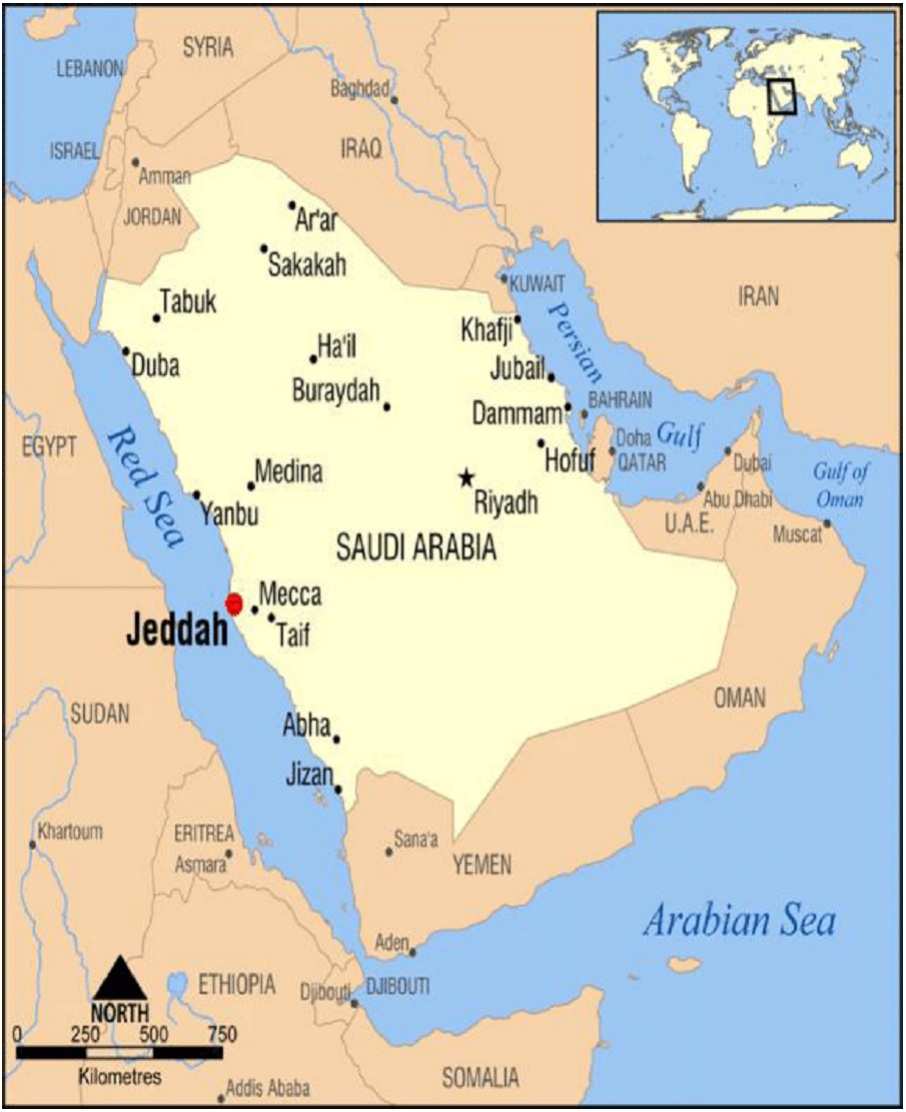
The study area. Map of the study area (Jeddah) from Saudi Arabia. Available via license: Creative Commons Attribution 4.0 International.

The study was conducted in Jeddah, Saudi Arabia. The city of Jeddah extends between Latitude:21.4858° North and Longitude: 39.1925° East (Fig. 1).

### Collection of sheep and goat blood samples

Data were collected as previously described in Metwally et al. (2021). Between March 2021 and June 2021, 299 blood samples (216 sheep and 83 goat) were randomly collected from slaughtered animals at The Municipal Abattoir of Jeddah, Saudi Arabia. Samples were collected from 244 males and 75 females. Animals were divided into two categories based on their age: young (less than one year) and adults (more than one year). 88 animals were adults (55 sheep and 33 goats), while 211 animals young (159 sheep and 52 goat). Animals subjected to the present study were either imported (from Sudan and Somalia) or locally bred in Saudi Arabia.

All samples were examined for blood parasites. Blood samples were collected in Vacutainer tubes containing ethylene diamine tetraacetic acid (EDTA) (BD Vacutainer^®^ Tube, Gribbeles Pathology, VIC, Australia) and were preserved at −20 °C for DNA extraction for PCR.

### DNA extraction and polymerase chain reaction (PCR)

Total genomic DNA (gDNA) was isolated using the DNeasy Blood& Tissue Kit (250) (Cat No./ID: 69506, Qiagen, Hilden, Germany) as dictated by the manufacturer’s protocol. All DNA samples were tested for the presence of apicomplexan (*Theileria* spp.) by PCR using primers targeting *18SrRNA* gene (460–520 bp), as described previously (Gubbels et al., 1999) (RLBF: GAGGTAGTGACAAGAAATAACAATA; RLBR: TCTTCGATCCCCTAACTTTC). The genes were amplified using a thermal cycler (Variety^®^ 96-well thermal cycler, model 9902, Biosystems, Waltham, MA, USA). This process was performed in 50 µL of a mixture containing 25 µL of DreamTac DNA polymerase Master Mix 2 × (Thermo Scientific™, UK), 1 µL of each primer, and 2 µL of DNA template. The reaction was brought to a total volume of 50 µL with PCR-grade water (Invitrogen, UK). All tests included positive and negative controls. The PCR cycling is: 95 °C for 600 s, 95 °C for 30 s, 52 °C for 30 s (×40), 72 °C for60 s, 72 °C for 420 s. The PCR product was analyzed using electrophoresis (100 V for 45 min) with 1.5% agarose gel that included 10 µL/mL Syber safe (Thermo Scientific™, UK), which had a Tris acetate EDTA buffer (Cat no. 17890, Thermo Fischer Scientific, Inc., Wilmington, DE, USA). The gel was photographed with a UV imaging system (Imagconat Laz4000, GE Healthcare Life Sciences, UK). The size of each product was estimated by comparing it with a 100 bp DNA ladder (Thermo Scientific UK, UK) (Alanazi et al., 2018a; Alanazi et al., 2018b).

### Nucleotide sequences of the *18S rRNA* gene and the phylogenetic approach

To verify the presence of *T. ovis*, positive samples were transported to the Central Lab, Female Students Campus, King Saud University, for sequencing of the *18S rRNA* region using forward RLBF and reverse RLBR primers. The results were compared with existing sequences in the NCBI GenBank. To verify the presence of *T. ovis*, positive samples were transported to the Central Lab, Female Students Campus, King Saud University, for sequencing of the *18S rRNA* region on a 310 Automated DNA Sequencer (Applied Biosystems, Foster City, CA) using BigDyeTM Terminator v3.1 Cycle Sequencing Kit (Applied Biosystems, Foster City, CA) using forward RLBF and reverse RLBR primers.

The sequences were processed using Geneious Geneious prime (2020) 2020.1.2 Build 2020-04-07 08:42 Java Version 11.0.6+10 (64 bit) (Geneious). As a pre-processing step, all sequences were truncated using the *error probability method,* with a limit of 0.05 on both sides. Related sequences were retrieved from GenBank after performing Basic Local Alignment Search Tool a (BLAST) (Altschul et al., 1990) search. Then, multiple sequence alignments were generated using CLUSTAL Omega (Sievers et al., 2011) implemented in the *Geneious* software. All the multiple sequence alignments used in this study are provided in the supplementary material in FASTA and Nexus formats. The phylogenetic tree was created using the neighbor-joining method (Saitou & Nei, 1987) implemented in MEGA 11 (Tamura, Stecher & Kumar, 2021). The evolutionary distances were computed using the Kimura 2-parameter method (Kimura, 1980) and 10,000 replicates.

### Statistical analysis

Data from sheep and goats infected with *T. ovis* were analyzed using the Chi-Square test and the SPSS statistical program version 26. The results are presented as numbers and percentages at a significance level of *p* ≤ 0.05.

## Results

### Prevalence of *T. ovis* infection in examined sheep and goats

This study, which was conducted on 216 sheep and 83 goat samples (Table 1), showed that the *T. ovis* infection rate was higher in goats (57.8%; 48/83) than in sheep (51.9%; 112/216). The infection rate was more significant in adult animals (63.6%; 56/88) than in young animals (49.3%; 104/211), and in females (61.3%; 46/75) than in males (46.7%; 114/244). Regarding animal breed, it was found that the infection rate did not differ significantly between the Sudanese and local Saudi animals, but the rate in both breeds differed significantly from that in the Somali animals.

### PCR amplification and nucleotide sequence analysis of the partial *18S rRNA* gene

**Figure 2:**
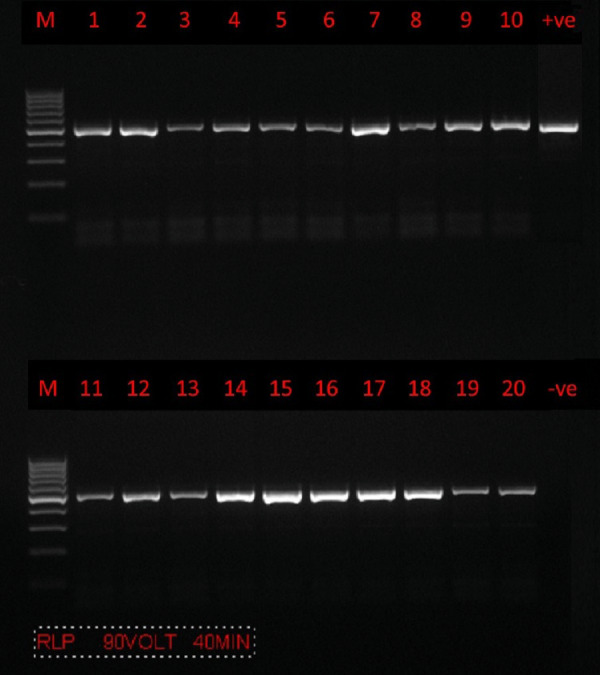
PCR amplification. Agarose gel (1.5%) electrophoretogram with a 100-bp DNA ladder. PCR analysis of the *18S rRNA* gene revealed a 500 bp band derived from *Theileria* spp. isolates from sheep (lanes 1–10) and goats (lanes 11–20).

PCR amplification was successfully carried out using only a set of primers (RLBF and RLBR) to amplify a 460–520 bp fragment of the *18S rRNA* gene from *Theileria* spp. (Fig. 2). A total of 160 positive sequences were analyzed in this study, of which 112 sequences were collected from sheep and 48 from goat. The sheep sequences were found to have a pairwise identity of 96.8% and a GC content between 34.2% and 51.7%. The length of the sheep sequences ranged from 416 nt–448 nt. The goat sequences were found to have a pairwise identity of 97.7% and a GC content between 37.6% and 53.2%. The length of the goat sequences ranged from 437 nt–432 nt. The inter-specific similarity of the sheep and goat sequences was 97.6%. The results of the BLAST search showed that the sequences are similar to the *Theileria* spp. of small subunit rRNA gene partial sequence reported in Turkey (accession numbers MW810474 –MW810486), with 84.4–99.8% pairwise identity and 52.35–99.79% coverage. A total of 12 sequences (six sequences from goats and six sequences from sheep) were randomly selected, analyzed, and deposited in GenBank. Accession numbers were assigned as follows: goat (MZ078474, MZ078473, MZ078470, MZ078469, MZ078465, and MZ078464) and sheep (MZ078475, MZ078472, MZ078471, MZ078468, MZ078467, and MZ078466).

**Table 1:** PCR-based prevalence of *T. ovis* infection detected in sheep and goats in this study.

**Trait** **factor**	**Infection**			**Chi-Square Sig.**
	**No.**	**%**	**Total no.**	
**Age**				0.000
Young (6–12 months)	104	49.3 b	211	
Adult (12–18 months)	56	63.6 a	88	
**Sex**				0.000
Male	114	46.7 b	224	
Female	46	61.3 a	75	
**Breed**				0.000
Somali	22	40.0 b	55	
Sudanese	67	52.7 a	127	
Local (Saudi)	71	60.7 a	117	
**Species**				0.000
Sheep	112	51.9 b	216	
Goat	48	57.8 a	83	

**Notes.**

The super-letters (a, b) are used to denote in which cases the percentage of infection differed significantly at *p* ≤ 0.05.

**Figure 3:**
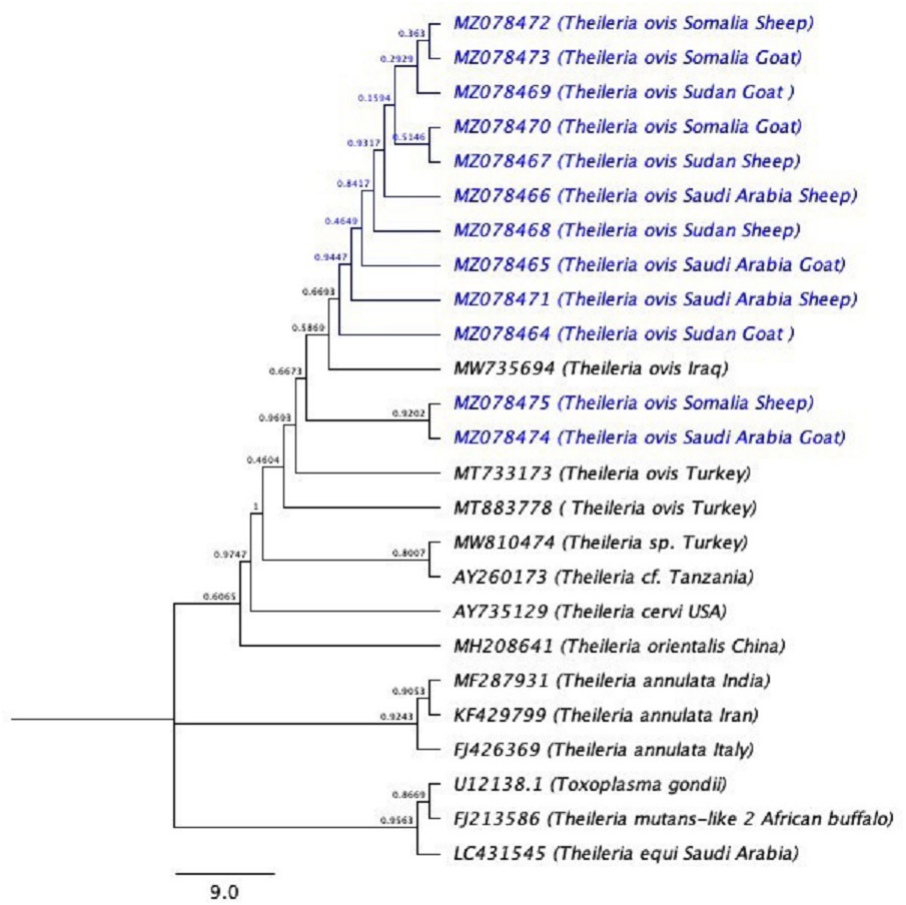
The phylogenetic tree using (*Toxoplasma gondii*) as the out-group. The phylogenetic tree inferred using the Neighbor-Joining method with *Toxoplasma gondii* is used as outgroup. The evolutionary distances were computed using the Kimura 2-parameter method (Kimura, 1980) and 10,000 replicates. The sequences of the current study are highlighted in blue.

The phylogenetic tree is shown in (Fig. 3). U12138.1 (*Toxoplasma gondii*) was used as the outgroup.

## Discussion

The hemoprotozoan *Theileria* spp. are responsible for significant levels of parasitic disease in the dairy industry in many countries, including Saudi Arabia. The tick-borne hemoprotozoans mainly infect cattle and small ruminants in tropical and subtropical regions, reducing their productivity (Mehlhorn & Schein, 1985). In the present study, *18S rRNA* gene partial sequences were used to evaluate the phylogenetic relationships between Saudi sheep and goat *T. ovis* strains and other *T. ovis* strains and different *Theileria* spp. Although the presence of *T. ovis* in Saudi Arabia is well known, and phylogenetic studies with *T. ovis* have been conducted in European countries, such studies have not been conducted in Saudi Arabia.

In the current study region, the prevalence of *T. ovis* is surprisingly high: 57.8% of goats and 51.9% of ovine herds. The high prevalence could be caused by the import or frequent movement of live ruminates from countries where *T. ovis* is endemic. The prevalence of *Theileria* spp. detected by PCR in this study is higher than previously reported in other localities in Saudi Arabia. For example, a prevalence of 19.9% and 6.9% of *Theileria* spp. in sheep and goats, respectively, was recorded in Al-Qassim Region of Saudi Arabia (Hussein et al., 1991). The prevalence of *Theileria hirci* in Al-Qassim Region was found to be 20.46% and 7.57% in apparently healthy sheep and goats, respectively (El-Metenawy, 1999). In addition, *Theileria* spp. infection rates of 5–24% in sheep and goats combined have been recorded in different provinces in Saudi Arabia (Al-Khalifa et al., 2009). Based on microscopic examination, 33.2% of sheep and 25.2% of goats were found to be infected with hemoprotozoan parasites, while PCR detected hematozoan infection in 46% of sheep and 33.7% of goats (Alanazi et al., 2019).

Studies in other countries have recorded lower prevalence rates of *Theileria* spp. infection. For instance, in Iraq, 22.8% of sheep were reported to be infected with *Theileria* spp. (using blood smears) (Dhaim, 2014). In Egypt, numerous studies have indicated the *Theileria* spp. infection rate in sheep and goats 15.6–87.5% (Hegab et al., 2016); Radwan & Kelesh, 2009). In Sudan, the *Theileria* spp. infection rate in preslaughtered animals was reported as 17.8% (Ahmed, 2016). Different rates of *Theileria* spp. infection have been reported in Iran: 36.17%, 18.6%, and 25.35% (Bahrami, Hosseini & Razmjo, 2013; Jovani & Tella, 2006; Razmi et al., 2006). In Turkey, the incidence of *Theileria* spp. in sheep and goats was 18.29% and 2.88%, respectively (Altay, Dumanli & Aktas, 2012). The variances in the above-mentioned prevalence rates may be due to differences in animal management and movement, and the environment in which the studies were performed. There may also be some differences in the results due to the sample size tested and the microscopic methods used. However, parasite prevalence estimates are often based on small sample sizes due to low numbers of hosts or difficulties associated with laboratory analytical methods (Jovani & Tella, 2006). Local weather conditions affecting tick distribution could also affect these results. Due to the duration of their off-host life cycles, ticks are extremely susceptible to climate change; processes such as development and survival depend on high temperatures and humidity (Sutherst et al., 1979). Therefore, the abundance of ticks is likely to show seasonal variation; it depends on the relative effects of temperature on development and mortality during the different developmental stages (Lindgren, Tälleklint & Polfeldt, 2000).

The present study showed a significant effect of sex on the prevalence of *T. ovis* infection. The rate of infection in females was 61.3% (46/75), and the rate in males was 46.7% (114/244). These finding are not in agreement with the results of several other studies (Alanazi et al., 2019; Iqbal et al., 2013; Khan et al., 2017; Naz et al., 2012; Razmi, Hosseini & Aslani, 2003). The contrasting of the findings may be due to high susceptibility of female to biting by ticks.

The current results also showed that the prevalence of *T. ovis* (*p* ≤ 0.05) was affected significantly by age. Most of the *Theileria* spp. infections were found in animals that were one year of age or older, since who live more will be more suspected to infestation with ticks. These findings are consistent with those of several previous studies (Alanazi et al., 2019; Dhaim, 2014; Guo et al., 2002; Naz et al., 2012). However Razmi, Hosseini & Aslani (2003) and Iqbal et al. (2013) reported that animals under one year of age had higher rates of infection than those over one year.

In the present study, *T. ovis* was found to be the only hemoprotozoan infecting sheep and goats using different species-specific primers. This is consistent with past molecular studies (Alanazi et al., 2019; El Imam et al., 2016; Khan et al., 2017).

In our study, one genetic marker (*18S rRNA*) for *Theileria* spp. in sheep and goats was used. All DNA samples of *Theileria* spp. during the PCR amplification of the partial *18S rRNA* gene yielded bands.

When blasting these sequences in GenBank, the *Theileria* spp*. 18S rRNA* sequences were found to be very similar to those in Turkey (accession numbers MW810474 –MW810486), with 84.4–99.8% pairwise identity and 52.35–99.79% coverage. To our knowledge, this is the first time that phylogenetic analysis of tick-borne pathogens has been conducted on small ruminants from Saudi Arabia.

## Conclusion

*Theileria* spp. infecting sheep and goats in Jeddah, Saudi Arabia, were identified as *T. ovis*. Phylogenetic approaches have confirmed for the first time that sheep and goats in Jeddah, Saudi Arabia, have been infected with *T. ovis*. The present study recorded that there was a significant effect of sex and age on the prevalence of *T. ovis* infection. As the rate of infection in females was higher than that in males and animals over one year of age had higher rates of infection than those less than one year.

## Supplemental Information

10.7717/peerj.12596/supp-1 Supplemental Information 1 Multiple sequence alignments in FASTA and NEXUS formats

## Additional Information and Declarations

### Field Study Permissions

The following information was supplied relating to field study approvals (i.e., approving body and any reference numbers): Deanship of Postgraduate Studies, University of Jeddah, Saudi Arabia.

### DNA Deposition

The following information was supplied regarding the deposition of DNA sequences: A total of 12 sequences (six sequences from goats and six sequences from sheep) are available at GenBank: goat (MZ078474, MZ078473, MZ078470, MZ078469, MZ078465, and MZ078464) and sheep (MZ078475, MZ078472, MZ078471, MZ078468, MZ078467, and MZ078466).

### Data Availability

The following information was supplied regarding data availability: A total of 12 sequences (6 sequences from goats and 6 sequences from sheep) are available at GenBank: goat (MZ078474, MZ078473, MZ078470, MZ078469, MZ078465, and MZ078464) and sheep (MZ078475, MZ078472, MZ078471, MZ078468, MZ078467, and MZ078466).
